# From silos to synergy: Integrating approaches to investigate the role of prior knowledge and expectations on episodic memory

**DOI:** 10.3758/s13423-024-02505-4

**Published:** 2024-05-01

**Authors:** Carla Macias, Kimele Persaud

**Affiliations:** Psychology Department, Rutgers University – Newark, Smith Hall, 101 Warren Street, Newark, NJ 07102 USA

**Keywords:** Episodic memory, Prior knowledge, Expectation-incongruent, Expectation-congruent, Computational models, Development, Adult behavioral approach

## Abstract

Significant progress in the investigation of how prior knowledge influences episodic memory has been made using three sometimes isolated (but not mutually exclusive) approaches: strictly adult behavioral investigations, computational models, and investigations into the development of the system. Here we point out that these approaches are complementary, each approach informs and is informed by the other. Thus, a natural next step for research is to combine all three approaches to further our understanding of the role of prior knowledge in episodic memory. Here we use studies of memory for expectation-congruent and incongruent information from each of these often disparate approaches to illustrate how combining approaches can be used to test and revise theories from the other. This domain is particularly advantageous because it highlights important features of more general memory processes, further differentiates models of memory, and can shed light on developmental change in the memory system. We then present a case study to illustrate the progress that can be made from integrating all three approaches and highlight the need for more endeavors in this vein. As a first step, we also propose a new computational model of memory that takes into account behavioral and developmental factors that can influence prior knowledge and episodic memory interactions. This integrated approach has great potential for offering novel insights into the relationship between prior knowledge and episodic memory, and cognition more broadly.

## Introduction

An important and long-standing question of cognition is: How do prior knowledge and expectations about the world relate to and influence the encoding and reconstruction of information in memory. Understanding the interaction between prior expectations and episodic memory has important implications for memory in real world contexts, such as eyewitness scenarios and optimizing learning in educational settings. As such, episodic memory research over the past few decades has made substantial strides in addressing this question using three sometimes isolated (but not mutually exclusive) approaches: behavioral investigations strictly with adults, computational cognitive modeling, and cognitive development studies with children. Behavioral studies with adults have helped to describe the processes that underlie memory for expectation-related (e.g., expectation-congruent and incongruent) information.^1^ Computational cognitive modeling has helped to explain the goals of the memory system in general, and in terms of the computations it operates over given system constraints. Likewise, tools of developmental psychology have helped to investigate the developmental origins, constraints, and potential change of processes involved in integrating prior knowledge in the memory system. Independently, each of these three approaches have been fruitful in advancing our understanding of the interaction of prior knowledge and memory (e.g., Stahl & Feigenson, 2017). Similarly, combining at least two of these approaches has also been informative in grounding theories of memory and knowledge interactions (e.g., Bein et al., 2015; Brod & Shing, 2019; Hemmer & Steyvers, 2009a, 2009b; Huttenlocher et al., 1991; Ortiz-Tudela et al., 2017; Sakamoto & Love, 2004, Sherman & Frost, 2000; see Bjorklund, 1987; Shing & Brod, 2016, for further discussion and review). Yet, the literature employing these approaches, although vast, has produced somewhat opaque theories and surprisingly mixed findings. Moreover, the contexts and domain dependencies that govern when one type of information is prioritized in memory over another is unclear. Despite the wealth of literature, important open questions regarding the interaction between prior knowledge, expectation-related information, and memory persist.

Thus, in this paper, we lobby for integrating all three approaches as a natural next step in advancing our understanding of the role of prior knowledge and expectations in episodic memory. Specifically, we point out that adult behavioral, computational, and developmental approaches are complementary. That is, the findings from one approach can provide a critical test of the theories and hypotheses gathered from the others. To illustrate, we first discuss each approach independently. We highlight past insights on the relationship between prior expectations and memory that have been garnered from each approach as well as limitations and open questions that remain. Next, we illustrate how some limitations and open questions can be addressed by integrating approaches. We then present a single case study that combines all three approaches. Finally, we present a new developmentally inspired model of memory that makes explicit predictions about how behavioral and developmental factors might influence prior knowledge and episodic memory across the lifespan. This integrated approach has immense potential for advancing our understanding of memory as an adaptive cognitive system.^2^

### Memory for expectation-related information

While there are a number of domains that could illustrate the benefit of integrating adult behavioral, computational, and developmental approaches, here we focus on memory and expectation-related (i.e., expectation-congruent and incongruent) information for several reasons. First, our prior knowledge and expectations about the world influence not only how we store and retrieve information from memory, but also every facet of cognition more broadly. As such, there is a wealth of research examining the role of prior expectations across a wide array of cognitive domains, including perception (Abreo et al., 2023; Alley et al., 2020), object recognition (Biederman et al., 1982; Hollingworth & Henderson, 1998; Torralba, 2003), category learning (Heit, 1998; Palmeri & Nosofsky, 1995; Sakamoto & Love, 2004), social cognition (Hastie & Kumar, 1979; Sherman & Frost, 2000; Stangor & Mcmillan, 1992), and cognitive development (Castel, 2005; Maril et al., 2011; Robertson & Kohler, 2007; Stahl & Feigenson, 2015, 2017). And in memory, in particular, this area of study extends across stimuli domains, including visual memory (Bae et al., 2015; Donkin et al., 2015; Persaud & Hemmer, 2014, 2016), lexical memory (Bein et al., 2015), event memory (Maril et al., 2011; Greve et al., 2019), and musical memory (Schmuckler, 1997). As such, understanding the role of prior expectations provides insight not only for memory research, but also for broader theories of cognition.

Second, characterizing the interaction between memory and expectation-related information can provide key insight into the representational structure of stored content in the mind (Hemmer & Steyvers, 2009a, 2009b; Persaud & Hemmer 2016; Sakamoto & Love, 2004). Understanding the role of expectations in memory touches on the broader point of how top-down knowledge (i.e., the representational nature of encoded and stored information) influences memory, connecting to general principles of cognition and learning. Third, while the literature on the interaction between memory and expectation-related information is quite dense, paradoxical findings persist and open questions regarding the psychological processes that underlie memory remain. Integrating methodological approaches to further investigate this domain has immense potential for adjudicating between theories and pushing our understanding of prior knowledge and memory interactions forward.

Finally, studies of memory for expectation-congruent and incongruent information have already employed all three approaches independently. Behavioral studies have characterized memory for expectation-related information in adults (Bein et al., 2015; Ortiz-Tudela et al., 2017; Sherman & Frost, 2000). Computational models have explained some of the processes and mechanisms that contribute to memory for expectation-related information (Hemmer & Steyvers, 2009a, 2009b; Huttenlocher et al., 1991; Sakamoto & Love, 2004). Developmental studies have explored how expectation-related information shapes memory and learning, particularly in children (e.g., Brod & Shing, 2019; Stahl & Feigenson, 2017; see Bjorklund, 1987, Shing & Brod, 2016, for further discussion and review). Open questions and future directions of these studies create prime opportunities for integrating approaches. Doing so promotes theory advancement and is a vital next step toward developing a unified account of the episodic memory system across sometimes independent methodologies.

In what follows, we first present the three approaches and highlight what we have learned about the relationship between prior knowledge and episodic memory from using them independently. We also detail some limitations and open questions that persist under each approach. We then present potential solutions that fall out of the integration of approaches. Next, we present a case study that leverages integration, and then present a new theoretical model that serves as an initial step for how integrating approaches can address existing open questions regarding the role of prior knowledge and memory. The goal of this paper is to demonstrate how the nuances of adult and child behavioral approaches can be used to design, constrain, and test modeling approaches. At the same time, modeling approaches can be leveraged to explore the processes and mechanisms that underlie behavior and developmental change. They can also inform the design of behavioral investigations, and interpretation of adult and child behavioral data. In doing so, we promote integrating approaches by presenting new considerations for how to leverage combining approaches to address limitations and remaining open questions.

## Methodological approaches: Past insights, limitations, and open questions

### Adult-behavioral approaches

Strictly behavioral studies with adults have been the hallmark of investigations into the intricacies of the memory system. Adult empirical studies have investigated a wide array of fundamental questions of human memory processes and have revealed a number of important memory phenomena, like the substantial role of prior knowledge. Behavioral methods are particularly useful for gathering evidence to test theories of cognition as well as rule out alternative explanations. As a result, the adult behavioral literature exploring how the mind makes use of expectation-congruent information and reconciles expectation-incongruent information is a vast area of inquiry (see Stangor & McMillan, 1992, Rojahn & Pettigrew, 1992, for meta-analyses and review). Despite this large literature, behavioral studies of memory for congruent and incongruent memoranda have been in some ways, limited, inviting opportunities for integrated approaches. Here we point out a few.

On the one hand, a benefit of the wide array of adult behavioral studies is that there has been an explosion of theories to better explain memory for congruent information (Bein et al., 2015; Craik & Tulving, 1975; Schulman, 1974), incongruent information (Fabiani & Donchin, 1995; Hunt, 1995; Rangel-Gomez & Meeter, 2013; Verguts & Notebaert, 2009), or both (Hastie & Kumar, 1979; Hastie, 1980; Greve et al., 2019; Sherman & Frost, 2000; Srull & Wyer, 1989; van Kesteren et al., 2012). On the other hand, a limitation of these studies is that because they are highly variable and lack a unified account of memory, it is difficult to decipher whether discrepant findings result from insufficient theories or simply differences in experimental stimuli and design. For example, conflict-based theories of memory suggest that studying information that conflicts with prior knowledge (expectation-incongruent items) selectively guides attention and memory resources, resulting in better memory for expectation conflicting information (Krebs et al., 2015; Rosner et al., 2015; Varguts & Notebaert, 2009). Yet, other studies that have sought to explore conflict-based theories using similar expectation-incongruent conditions, failed to find support for better memory for conflicting items (e.g., Ortiz-Tudela et al., 2017, 2018). As a result, it is unclear whether conflict in the form of expectation incongruence always produces better memory, if the boost to memory is only observed for certain kinds of conflicts, or certain degrees of conflict. Moreover, it is unclear what unifying goal of the memory system is being served by prioritizing expectation conflicting information in general.

A similar discrepancy was found in studies on how prior expectations in the form of schemas influences recognition memory during category learning (De Brigard et al., 2017; Sakamoto & Love, 2004; Yin et al., 2019). While some research found better recognition memory for schema incongruent items because of the violation of rules inherent to the schema structure (e.g., Sakamoto & Love, 2004), other research found better memory for schema-congruent items, but also an increase in false alarms (e.g., De Brigard et al., 2017; Yin et al., 2019). In further contrast, other studies found better memory for both schema-incongruent and congruent information, depending on cognitive load demands during encoding (Sherman & Frost, 2000) or the presence of an explicit prediction error (Brod et al., 2018; see Stangor & McMillan, 1992, for a comprehensive list of other factors). These mixed findings illustrate that although strictly behavioral studies are extremely informative and have advanced our understanding of memory and prior expectation interactions, there remains no clear consensus under which conditions memory prioritizes certain kinds of expectation-related information, and more importantly, what unifying goal of the memory system is being served in prioritizing certain information across contexts (see van Kesteren et al., 2012, in the domain of Cognitive Neuroscience for a similar argument). Instead of a comprehensive picture of the relationship between memory and expectations, we are left with an incomplete paradox that warrants further investigation (Souza et al., 2022). Integrating across methodological approaches might help to bring clarity by offering sophisticated machinery and toolkits for specifying theories, generating predictions, and performing both qualitative and quantitative comparisons of competing theories and their fit to empirical data.

Second, given the strong association between prior knowledge and expectation-congruent information, behavioral studies have been tasked with differentiating between responses that are more likely to reflect retrieved memories from those that reflect informed guessing using prior knowledge (De Brigard et al., 2017; Yin et al., 2019). For example, imagine that an individual is tasked with freely recalling objects from a typical kitchen scene. If participants produce responses for objects that are prototypically found in kitchens, it is difficult to determine whether the response is truly evidence of a retrieved memory or a guess from prior knowledge of objects associated with kitchens. To address this concern, behavioral studies often need to compute accuracy for expectation-congruent items as the difference between correct recall of truly studied items (hits) and recall of items that are congruent with the study context (lures) but have not been studied (false alarms) (e.g., Höltje et al., 2019; van Kesteren et al., 2013). While this is a reasonable approach to evaluating accuracy, it can only tell us about memory for congruent items over the aggregate of produced responses. This leaves opportunities for characterizing the accuracy of expectation-congruency at the individual item level, where differences at this level can be informative to our understanding of memory. To foreshadow, computational cognitive models have been developed to demarcate responses that are likely to reflect true memories from those that reflect guesses at the individual trial level and would be useful for corroborating and building on strictly empirical findings. Taken together, these limitations readily lend themselves to integrated approaches.

Third, much of what is known about memory for expectation-related information is based on memory studies with young adult participants. As mentioned earlier, these studies identify important cognitive factors that influence memory for expectation-related information across contexts. However, far less research explores the interactions between memory and expectations across development. Understanding memory for expectation-related information in development creates interesting opportunities to explore whether integrating prior expectations is a foundational memory process or if it is a process that only results from increases in age (see Bjorklund, 1987; Brod et al., 2013, for a discussion). Understanding general mechanisms of memory that persist across development versus those that require maturation before coming online might help to further elucidate why some factors influence memory for expectation-related information in certain contexts.

In sum, the purpose for pointing out these potential limitations of strictly behavioral studies with adults is not to suggest that this methodological approach is in any way problematic or any less informative for this area of inquiry, but rather to suggest that they create occasion for integrating their findings with other methodological approaches to advance our understanding of this cognitive domain.

### Computational modeling approaches

A second methodological approach for investigating memory for expectation-related information, and memory in general, is computational cognitive modeling. Computational approaches provide powerful machinery for defining, testing, and validating theories of cognitive behavior, and memory more specifically. These approaches allow for the specification of the goals, constraints, and processes that underlie cognitive systems (Leider & Griffiths, 2020; Sims et al., 2012). Next, we detail some of the past insights that have been garnered from taking computational approaches to investigate expectations and memory and then present opportunities for further model development by integrating approaches.

Computational cognitive models, and rational models in particular, have been instrumental in elucidating the role that prior knowledge and expectations play in episodic memory (Bae et al., 2015; Cibelli et al., 2016; Donkin et al., 2015; Hemmer & Steyvers, 2009b; Hemmer et al., 2015; Huttenlocher et al., 2000; Persaud & Hemmer, 2014, 2016; Steyvers et al., 2006; Xu & Griffiths, 2010). Past research employing computational approaches has revealed that the degree to which prior knowledge influences memory depends on the degree of uncertainty or noise in the stored memory representations. That is, the better information is initially encoded into memory, (i.e., greater fidelity and less noise), the less influence prior expectations will have on how that information is later recalled and vice versa (Bae et al., 2015; Donkin et al., 2015; Huttenlocher et al., 2000; Persaud & Hemmer, 2014, 2016). These models have also found that prior knowledge can be hierarchically organized and exert strong influences on memory at multiple levels of abstraction (Hemmer & Styevers, 2009b; Hemmer et al., 2015; Robbins et al., 2014).

Theoretically this modeling framework can capture memory for both expectation-congruent and incongruent items (see van Kesteren et al., 2012; Zhang, 2022), yet far less computational work has modeled the latter type of expectations. Modeling memory for incongruent items within these frameworks is particularly complex because it is not always clear how to formalize the categories to which expectation-incongruent items belong (see Zhang, 2022, for a similar point). For example, imagine modeling memory for incongruent colors of objects (e.g., a blue banana). On one hand, the blue banana might be treated as an atypical member of the banana category. According to rational models of memory, when the initial memory trace for the banana becomes noisy, recall will regress toward a more prototypical color for banana. Yet, recent empirical evidence suggests that this is not always the case. Tompary & Thompson-Schill (2021) found that memory for items that fall near the boundaries of categories are not influenced by the overall category (e.g., do not regress toward the mean). It is possible that the blue banana is not classified as an atypical member of the category, but instead is encoded as a new category of banana which might explain the lack of resemblance to the prior at recall (Sakamoto & Love, 2004). But what determines when an expectation-incongruent item is classified as atypical or belonging to a new category? How might these different treatments of expectation-incongruent items be instantiated within rational accounts of episodic memory? If such an account of category treatment of incongruent items were formally specified, what kinds of stimulus domains would be relevant for testing the robustness of these models? In other words, are all types of expectation-incongruent items represented in the same way? Also, are they represented in the same way across different memorizers? Addressing these questions would provide interesting challenges and opportunities for expansion of current rational models of expectations and memory. To foreshadow, we propose a new rational model that offers a different theoretical treatment of expectation-incongruent items and might better capture performance when memory for these items deviates from predictions of existing models (see *New directions* section).

Second, computational models have been useful for adjudicating between competing theories of how knowledge and expectations are represented in the mind and the downstream effect this structure has on memory and learning. For example, Sakamoto and Love (2004) implemented and compared three models each making different assumptions of how expectation-related information is structured in the mind. The first model assumed that congruent and incongruent information are stored separately based on a strict rule (i.e., RULEX). The second model assumed that expectation-related information was stored in clusters, such that similar items are stored together, and maximally dissimilar items are stored as new clusters (i.e., SUSTAIN). The third model assumed that every studied item was stored separately forming its own context and episodic trace. They found that a cluster-based account (i.e., the SUSTAIN model) provided the superior fit to memory data, suggesting that representations of category knowledge and expectations are more likely to be structured in a schema-like (i.e., cluster) than rule-based fashion, thereby successfully adjudicating between theories.

This exciting work captures how adults might represent expectations in the mind and use those expectations during learning and memory and opens up further questions related to this modeling approach. For example, across different learning contexts, how does a modeler decide which representational structures should be under consideration? Are our underlying representations always fixed or flexible throughout the course of learning? Are there context dependencies or cognitive dependencies that dictate when we might use one representational structure for prior knowledge over another? One method for addressing these open questions within this modeling framework, and modeling more broadly, would be to consider a broader range of adult behavioral studies as well as developmental studies (see Integrating approaches for more detail).

Third, prior expectations, and how they are brought to the task of remembering can vary across stimulus contexts (e.g., Brod et al., 2018), memory tasks (Sherman & Frost, 2000; van Kesteren et al., 2012), and across groups of memorizers (e.g., Brod & Shing, 2018; Persaud et al., 2021). For example, prior knowledge differentially impacts memory performance as a function of whether participants are required to make a judgment before or after seeing study memoranda (Brod et al., 2018). The impact of expectations on memory also depend on additional task-related factors, such as increased cognitive load and whether a recall versus recognition task is employed (Sherman & Frost, 2000; Stangor & McMillan 1992), and the distribution of expectation-congruent and incongruent items on the study list (Morita & Kambara, 2022). Further, there are also instances where although an individual has semantically rich prior knowledge, that knowledge might not be accessed by the memorizer, thereby having very little impact on the recall of information from memory (e.g., Bransford & Johnson, 1972). This distinction between having prior knowledge versus accessing that knowledge such that it has downstream effects on memory was articulated in Brod (2021) taxonomy of prior knowledge. Yet, most current model instantiations are predicated on behavioral data taken from standard memory tasks that do not vary on many of these important factors. Additionally, the data are often based on studies with young adults and use very limited stimulus domains. A test of the robustness of our current models would be to evaluate how well they capture memory across a wider array of expectation-related stimuli, experimental task designs, and diverse groups. Doing so allows for the development of a more comprehensive picture of the role of expectations within the memory system. To this end, here we propose going beyond the standard practice of designing models fit to arbitrary and semantically limited stimuli, by considering expectation-congruency and developmentally relevant factors that shape the memory processes.

Overall, computational cognitive modeling has been influential in characterizing the interaction between prior knowledge and memory. This approach sheds light on how expectations might be represented in the mind, how the structure of those representations bears distinct signatures on recalled memories, and how prior expectations and stored episodic traces interact during recall as a function of uncertainty. Yet, several interesting and open questions persist. Specifically, questions regarding how to model memory for expectation-incongruent items, how to capture variability in the ways expectations might be represented, and how to consider different expectation contexts during model parameterization. While of course these models already integrate behavioral and computational approaches, here we point out the necessity for future models of expectation-related memory to integrate a wider array of behavioral data, along with developmental data to further optimize computational cognitive models and provide a more comprehensive understanding of interactions between expectations and episodic memory. We return to this point in the *Addressing limitations of cognitive models* section.

### Developmental approaches

A third methodology for investigating memory for expectation-related information is developmental approaches. Developmental approaches provide unique insight for understanding the growth, maturation, and change in cognitive processes across the lifespan. In children, developmental research can help pinpoint the onset and incremental change of processes and mechanisms that underlie memory, including how memory is impacted by prior knowledge. In older adults, developmental work highlights the adaptive nature of the memory system in response to maturation, and in some cases, degradation (Carr et al., 2015; Dennis et al., 2008; Naveh-Benjamin, 2000). Furthermore, developmental research allows us to test the robustness of our theories and conceptualizations of cognition that have been conceived from studies primarily from young adults (e.g., WEIRD-Western, Educated, Industrialized, Rich, and Democraticundergraduates). While we have gained quite a bit of knowledge of what interactions between prior knowledge and expectations and memory look like in development, there are limitations and open questions that could be addressed using more integrative approaches.

First, we have learned from developmental research that the use of prior knowledge to inform memory is a ubiquitous process, extending across developmental groups (Brod & Shing, 2019; Duffy et al., 2006; Stahl & Feigenson, 2017, 2015). Children and even really young infants’ memories are shaped by their attention to expectation-incongruent information in their environments (Stahl & Feigenson, 2015, 2017). For instance, Stahl and Feigenson (2015) presented infants with a physical violation of expectation event (VOE; e.g., a rolling car passing through a solid wall) and found that infants better remember novel information associated with VOE objects. In another study, researchers found that young children better remember novel labels associated with objects that violated an established expectation (e.g., an object hidden in one location is found in another) relative to labels of objects that were consistent with an established expectation (e.g., an object hidden in one location is found in the same location – Stahl & Feigenson, 2017). Taken together, this research suggests that babies' and young children’s expectations about objects’ core physical principles (e.g., object solidity and spatiotemporal continuity) facilitate their memory for VOE object properties.

This exciting line of research presents numerous follow-up questions that are open for investigation. For instance, are young children’s memories influenced by different types of expectations, including less entrenched ones like schema-related expectations? What aspects of various types of events (e.g., VOE and Non-VOE) do children represent in memory and why? How do they reconstruct these events from memory? And finally, what is the fidelity in which expectation-related information gets encoded in children’s memory and how does it change over time? While developmental research alone has done a great job of tackling questions of memory and expectations in children, computational approaches, and theories from adult behavioral studies, could be instrumental in helping to address open questions that remain.

Second, we’ve also learned from developmental research that despite the ubiquity of using prior knowledge in early childhood, there is some additional processing of expectation-congruent information that older children can do that younger children cannot. This is evidenced by age-related increases in memory for congruent items but no age-related differences in memory for incongruent items (Geis & Hall, 1978; Ghatala et al., 1980; see Brod et al., 2013, for further discussion). For instance, in a study by Ghatala and colleagues (Ghatala et al., 1980) 8- to 14-year-old children were prompted to answer 36 questions (e.g., Is this a flower?) about words that were either congruent (e.g., “rose”/yes) or incongruent (e.g., “submarine”/no) with the question, and they had to recall the words after. They found a linear increase in recall accuracy with age for the congruent condition and no change in accuracy in the incongruent condition. The authors attributed this age-related difference in recall to older children’s ability to make use of an elaborative encoding process (levels-of-processing; Craik & Lockhart, 1972), which is supported by a wider and more established network of prior knowledge. This finding opens up several interesting questions. First, at an algorithmic level, what are the steps involved in the elaborative encoding process? What mechanisms support older, but not younger children’s ability to engage in elaboration? Lastly, why is the elaborative process facilitative of memory for congruent, but not incongruent items? Computational models might be instrumental in formalizing this elaborative encoding process and teasing apart various mechanisms that might be leveraged by older, but not younger children when encoding and retrieving expectation-related information from memory.

Third, findings from developmental research suggest that the influence of prior knowledge on memory across the developmental lifespan is not linear, but parabolic. That is, when children have well-established prior knowledge, they tend to rely heavily on their priors, in a manner that is different from young adults, but akin to older adults (Brod & Shing, 2019). For instance, in a recent study, Brod and colleagues presented participants, in three different age groups (6–7 years, 8–22 years, and 64–74 years), with familiar object and scene pairs that were either congruent (e.g., Tractor/Farm) or incongruent (e.g., Tractor/Ocean) with their established expectations. Following the initial presentation of object and scene pairs, participants completed a recognition memory task. Overall, all three age groups displayed better memory for the congruent object scene pairs relative to the incongruent pairs. However, they found that relative to young adults, older adults, and to a lesser extent younger children’s memories were more heavily biased by prior knowledge. This age-comparative study illuminates important nuances in how prior knowledge impacts memory. The finding of a U-shape as opposed to the linear influence of expectations on memory, stands in contrast with previous studies that show the tendency to rely on and be biased by schema-based knowledge increases with age (Metzger et al., 2008). This work opens interesting questions about the compensatory role of prior knowledge in memory. For example, given the variability in constraints that impact young children and older adults, what kinds of age-related and/or cognitive deficits might prior knowledge be leveraged to compensate for in recall? What kinds of task demands inhibit the compensatory role of prior knowledge in memory for young children? How do we disambiguate children and older adults’ accurate memories, where prior knowledge is leveraged, versus guesses using prior knowledge? Furthermore, in situations where children have sufficient prior knowledge, what conditions dictate whether that knowledge is accessible, and can be utilized to aid memory (see Brod et al., 2013; Brod, 2021, for a similar point).

Overall, developmental approaches have expanded our understanding of the interaction between prior knowledge and memory by providing a more comprehensive account of memory as it considers changes in factors (e.g., cognitive capacity) across the lifespan. This approach has demonstrated that while the influence of prior knowledge on memory is ubiquitous and occurs early in life, age-related differences, among other factors, can impact the degree to which prior knowledge is accessible and influences memory. This leaves a rich set of open questions to be explored. Namely, questions regarding the types of VOE that influence children’s memory, the aspects of VOE and Non-VOE events that are represented in memory (and why), the fidelity of expectation-related information over time, and the underlying processes and mechanisms that support the use of prior knowledge at different stages of development. Integrating approaches will further our understanding of the intricacies and complexities of all the factors that influence memory across the lifespan.

## Integrating aproaches: Addressing limitations and open questions across approaches

Previous investigations that have integrated across multiple methodologies in general have been fruitful in advancing the discussion of how expectations influence memory. As illustrated above, some investigations have combined computational modeling with adult behavioral studies, or child behavioral studies with adult behavioral studies. Yet, far less research has sought to combine all three approaches to investigate the complex interactions between prior knowledge and episodic memory. Here we suggest that integrating all three approaches, not only addresses limitations of each methodological approach alone, but also collectively can reconcile disparate findings in the literature and opens new avenues of inquiry. In what follows, we first revisit some of the limitations and open questions discussed earlier and how they might be addressed by integrating approaches. We then present a single case study and a new model as a launching point to highlight what important advances can be garnered from combining all three approaches for studying memory.

### Addressing limitations of behavioral studies with adults

While adult behavioral studies have informed our understanding of expectations and memory, there are some limitations of strictly behavioral approaches that can be addressed by integrating other approaches. As mentioned previously, adult behavioral studies of memory can widely vary, and mixed findings often make it difficult to evaluate whether differences result from underspecified theories or simply differences in experimental methodology. Further, ambiguities can arise in the interpretation of behavioral data that often warrant relatively sophisticated techniques for fine-grain analyses. Finally, behavioral memory data comes from relatively limited participant pools, most of which are predominantly young adults, calling into question how much of our findings of memory generalize to more diverse groups of memorizers.

To this end, integrating computational and developmental approaches might help to overcome some of the limitations of strictly behavioral investigations with adults. First, computational approaches can build on existing behavioral findings in two important ways. Computational approaches provide constraints on cognitive theories by setting expectations about the goals of the system and providing a framework for testing assumptions of those theories. Constraining the wide array of empirical theories of memory for expectation-related information and recasting them with respect to the goals of the memory system might help to reconcile mixed and paradoxical findings. A similar approach has been employed recently in the cognitive neuroscience literature to understand mixed findings of memory for expectation-related information with respect to neural mechanisms (Gilboa & Marlatte, 2017; Greve et al., 2019; van Kesteren et al., 2012; see Brod et al., 2013, for further discussion). Here we suggest that computational cognitive models might also help to shape the discussion.

Second, computational modeling can also facilitate a more in-depth analysis of memory for expectation-related information. As mentioned previously, a limitation of adult (and child) behavioral studies is that they often grapple with characterizing memory for expectation-congruent information to determine when responses reflect accurate memories versus guessing with prior knowledge. There have been a host of computational cognitive models developed to parse out responses that likely reflect memory traces from those that reflect guessing, even at the individual trial level (Donkin et al., 2015; Lew et al., 2015; Persaud & Hemmer, 2016; Zhang & Luck, 2008). This modeling approach might help to explicate mixed findings of memory from strictly behavioral studies, especially for expectation-congruent information.

Furthermore, developmental approaches can also shed light on the factors that influence memory processes. Development provides a good test of the mechanisms that are theorized to explain memory performance. In addition, with respect to change, developmental research can provide insight into the causal and emergent properties of memory that shape performance. Moreover, while it is well known that prior knowledge and expectations influence the encoding, storage, and retrieval of information from memory, it is less clear the contexts in which this influence is observed, and the factors involved in producing this effect. For example, does the use of knowledge and expectations to inform memory come online based on how much prior knowledge an individual has accumulated about study memoranda, based on the need to overcome issues of encoding, or issues of retrieval, or all of the above? These are often difficult questions to answer based strictly on adult behavioral studies where prior knowledge (except in the case of experts), general encoding ability, and retrieval ability are comparable. Instead, integrating developmental approaches (i.e., conducting studies across different age groups) might be useful because differences in the amount of prior knowledge and memory ability naturally vary, providing an ideal context to address these challenging questions.

Taken together, computational and developmental approaches can help overcome the limitations of basic adult behavioral research. While leveraging computational approaches to understand behavioral investigations of memory is nothing new, here we suggest that combining modeling and development has immense potential for further elucidating the role of prior knowledge and expectation-congruency for a more comprehensive understanding of episodic memory.

### Addressing limitations of computational models

While implementing computational cognitive modeling to understand episodic memory is a valuable tool, there are some challenges and limitations to general models^3^ and expectation-related models of memory that might be assisted by integrating other approaches. First, for practical reasons, many general models are designed to characterize memory for relatively limited, often unidimensional kinds of arbitrary study information. That is, the behavioral data on which many models are tested, assess memory for arbitrary stimuli, such as arbitrary pairings of colors to objects, arbitrary object locations, orientation, random word lists, etc. While this is a strength of this approach because it allows for the development of theoretically robust models, the findings from studies of how memory outcomes are impacted by a wider range of real-world, semantically rich information taken into the system are often underutilized (see Steyvers & Hemmer, 2012; Hemmer & Persaud, 2014, for a discussion). As a result, one general limitation is that it is unclear how the information we learn about memory from these computational approaches, such as the precision of memory over time, the allocation of memory resources, the rate of interference, the rate of guessing, etc., change as a function of the classes of study information being modeled.

In addition, current models are overwhelmingly designed to fit data from young adults, calling into question whether and how they might be optimized to capture performance across a wider array of memorizers (e.g., young children and older adults). More careful considerations of incorporating findings from development could entail modifying current parameters, incorporating additional parameters, or developing new models to accommodate performance across the lifespan. As such, there is a missed opportunity in current computational approaches for further model development and expansion, and in capturing the complexity of cognition more broadly.

The issue of current models being applied to limited kinds of behavioral data and data mostly from young adults is further exacerbated for models of memory seeking to explain the role of prior expectations. First, the expectations that individuals bring to the task of remembering can vary substantially. These expectations are semantically rich, they can be pre-existing or experimentally derived, they can vary in the degree to which they are congruent or incongruent with study memoranda, and importantly, they can differ across memorizers. It is likely that this diversity of expectations and their congruence with study information will substantively impact the memory outcomes that current models seek to quantify. They also might introduce additional constraints that might impact the interactions between expectations and memory that current models do not capture, particularly where it concerns constraints that influence memory in development (e.g., working memory capacity, inhibitory control, representational change, etc.).

To this end, integrating a wider range of adult behavioral studies and developmental approaches creates opportunities to test the robustness of and expand current models of memory. Strictly behavioral studies with adults and somewhat with children have systematically investigated memory for a wide variety of expectation-related information (see *Methodological approaches* section) across different stimulus domains, across different time points, using different encoding and retrieval tasks, and imposing different kinds of task demands. Additionally, cognitive development is a natural domain for quantifying cognitive constraints (e.g., inhibitory control, working memory capacity, etc.) that have downstream effects on memory and learning (Brod et al, 2020, Davidson et al., 2006). All these factors are likely to be informative for the kinds of representations considered in current models, the variables parameterized, the processes captured by model algorithms, and the overarching computational assumptions of goals and constraints of the memory system. Taken together, the rich, complex behavioral literature exploring the interactions between expectations and memory creates prime opportunities to challenge the robustness of current models of memory and optimize them given new insights.

Moreover, not only does an integrative approach address some of the limitations of cognitive modeling generally, but they also provide methods for reconciling limitations and addressing open questions for expectation-related models more specifically. For example, as previously mentioned, there is not a great deal of computational work characterizing memory for expectation-incongruent items. Also, memory for expectation-incongruent items does not always behave in ways predicted by current expectation-related models of memory (Tompary & Thompson-Schill, 2021). This presents challenges for how best to flexibly represent certain expectation-incongruent items within these models. It also engenders open questions related to modeling memory and expectation-incongruence. For instance, what are the domains that govern when incongruent items behave one way versus another? How might the models be optimized to capture this variability? In this instance, considering theoretical treatments of atypical/expectation-incongruent items in adult and child behavioral literature might be informative for thinking about how incongruent items should be formalized in models.

Finally, the power of any theory or modeling approach comes from the degree to which it can speak to and account for the variability in behavior. In general, adult memory literature and cognitive development supplies a rich source of variability in representation, constraint, and mechanism. Given that many modeling frameworks seek to specify representation, build in constraints, and illuminate mechanisms that underlie the processes of memory, capturing performance from a broader range of adult-behavioral studies and developmental studies provides an informative method for testing the flexibility of such models. In this way, understanding how computational problems can inform and be informed by behavioral investigations of memory in adults and children for expectation-related information is an important area of inquiry.

### Addressing limitations of developmental approaches

Developmental behavioral studies have been instrumental in understanding the complex relationship between prior knowledge and episodic memory. Yet, there are a few challenges with conducting strictly developmental work, especially when investigating prior knowledge and episodic memory, that might benefit from the integration of adult behavioral and computational approaches. As previously mentioned, developmental data is highly variable. Oftentimes, the variability we find in developmental data is a double-edged sword. On the one hand, variability is important because it highlights the complexity of factors that contribute to cognitive performance, consequently creating opportunities to explore how a wide range of mechanisms impinge on processes that underlie memory and cognition. On the other hand, however, the discrepancies found in highly variable data can be challenging for strictly behavioral approaches to independently reconcile. In this instance, considering other approaches might help. For example, computational approaches can provide a principled way to explore and reconcile the noise in developmental data as well as generate explicit predictions about what factors are contributing to the noise observed in performance. Predictions generated by computational models can then be leveraged to help design developmental studies and interpret developmental data. Computational modeling can also facilitate a more in-depth analysis to make sense of noisy memory data by capturing computational factors, like the fidelity of information over time (Bays et al., 2009; Brady et al., 2013; Donkin et al., 2015; Zhang & Luck, 2008), the allocation of precision resources to studied information (van den Berg, Shin et al., 2012), evaluating differences in the cost of errors associated with different kinds of information (Sims et al., 2012; Sims, 2016), and assessing the contribution of guessing and interference to memory performance (Bays et al., 2009; Lew et al., 2016).

Second, collecting developmental data can be costly (Schott et al., 2019). As a result, researchers often end up with small sample sizes and small effect sizes. It is well documented that there is a data problem in developmental research where datasets are limited in participant composition (sampling bias; Nielsen et al., 2017) and quantity (Frank et al., 2017; Schott et al., 2019). Behavioral work with adults and computational approaches might be useful for addressing this limited data problem. First, behavioral approaches with adults can provide historical perspectives to inform theory. They also bring access and understanding from larger datasets to better develop and evaluate theory, particularly theories that are also explored in developmental contexts. Similarly, computational models allow for the simulation of data to further develop falsifiable hypotheses regarding behavior (Wilson & Collins, 2019). In turn, simulated data based on well-defined theories and predictions can be compared to smaller developmental datasets, to further illuminate underlying mechanisms and processes of cognition.

It should be noted, explanatory work that points to universal mechanisms for memory may not be as negatively impacted by such sampling biases, but as we come to understand expectations in memory, it is important to recognize the ways in which culture and context as well as early adversity might impact the representation, interpretation, and development of these processes. Importantly, computational approaches such as model-based multiple imputation can help explore the degree to which behavioral results might be expected to generalize (e.g., see Yu et al., 2020).

Integrating approaches can also help address some of the limitations and open developmental questions related to the role of prior knowledge in memory. As mentioned earlier, one limitation is that it is often difficult to determine the underlying processes that drive age-related differences in memory for expectation-related information. For instance, Ghatala and colleagues (Ghatala et al., 1980) attributed age-related differences in recall of expectation-congruent information to older children’s ability to make use of an elaborative encoding process (levels-of-processing; Craik & Lockhart, 1972). But it is unclear what this elaborative encoding process entails. Therefore, some open questions remain. That is, what steps are involved in the elaborative encoding process? Which mechanisms support this elaborative encoding process in older children relative to younger children? Why is the elaborative process facilitative of memory for congruent, but not incongruent items? Computational models might help to formalize this elaborative encoding process and tease apart various mechanisms that might be leveraged by older, but not younger children when encoding and retrieving expectation-related information from memory. Also, adult behavioral approaches can offer a baseline for theoretical predictions when exploring these questions of memory in children. In this way, convergence between computational models, adult behavioral and developmental data is not only a powerful tool for validating and refining theories of memory and cognition (see Bejjanki & Aslin, 2020; Persaud et al., 2020, for a discussion) but also for uncovering the factors that impact memory performance across development.

In all, computational and strictly adult behavioral approaches can help overcome the limitations of developmental approaches in many ways. While it might come as no surprise that developmental research could benefit from integrated approaches, the use of modeling is not as pervasive. It is also not a theoretical approach that is often considered. Thus, we suggest that integrating all three approaches is advantageous for developing a comprehensive understanding of memory and the role of prior expectations.

In this section, we have highlighted both limitations and open questions stemming from each disparate approach and pointed out potential integrative solutions for addressing those limitations. While we acknowledge that incorporating computational modeling to understand behavioral data, particularly, adult data, is nothing new (e.g., Anderson, 1996**;** McClelland, 2009; Shiffrin & Steyvers, 1997), using modeling to inform development and development to inform modeling is not an approach that is widely employed. Here we suggest that we can optimize models by considering constraints learned from developmental research and a wider array of behavioral studies. We can also leverage computational approaches to facilitate the design of behavioral studies and fine-grain interpretation of behavioral data from both adults and children. In what comes next, we illustrate this point further by providing a single case study in which all three approaches were used to explore how adults and children reconstruct expectation-congruent information from memory.

## Illustrative example: Benefits of integrating all approaches

Above, we present opportunities for intersectionality between adult behavioral, computational, and developmental approaches. We suggest that integrating all three approaches allows memory researchers to address some of the limitations associated with each approach alone, as well as a method to start tackling questions that remain. One such example of integration of all three approaches is a recent paper that sought to understand how prior knowledge and episodic memory interact across developmental groups (Persaud et al, 2021). While previous evidence suggested that adults (Persaud & Hemmer, 2014, 2016) and children (Duffy et al, 2006) alike integrate prior knowledge during recall, the degree to which this integration occurs across groups was unclear. Furthermore, alternative theories could also explain behavioral patterns that look like the integration of prior knowledge. Yet, which competing theory best explained memory performance across groups was not formally evaluated.

**Fig. 1:**
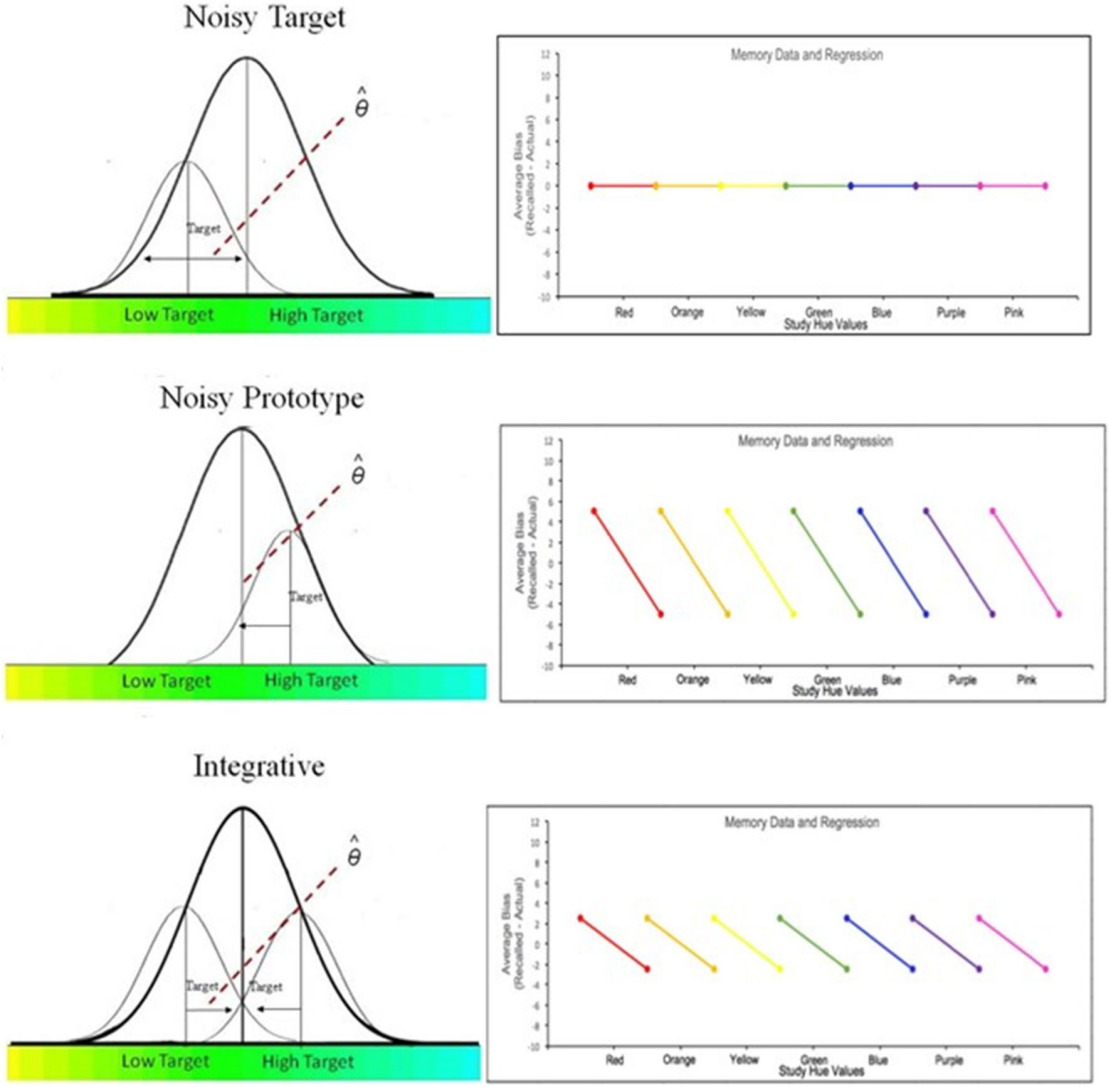
Idealized predictions for each process. The figure is replicated from Persaud et al. (2021). Idealized predictions for each process. ϴ is an estimate of a response under each strategy. **Left panels:** the larger curves are distributions over the colors that belong to a given color category centered over the prototype of the category. The smaller curves are distributions centered over target values and denote the direction of where color values are likely to be recalled in response to the target as prescribed by the different models. **Top left panel:** prediction for response distribution under the Noisy Target process. **Top right panel:** idealized qualitative prediction of recall bias by category under the Noisy Target process. **Middle left panel:** prediction for response distribution under the Noisy Prototype process. **Middle right panel:** idealized qualitative prediction of recall bias by category under the Noisy Prototype process. **Bottom left panel:** prediction for response distribution under the Integrative process. **Bottom right panel:** idealized qualitative prediction of recall bias by category under the Integrative process

To address this, Persaud and colleagues (Persaud et al., 2021) implemented a behavioral task where both adults and preschoolers studied and recalled the color of several unique shapes. To control for the common confound between age and prior knowledge, the authors established that both adults and children had similar knowledge and expectations about colors and color categories. Overall, they found that both adults and children’s recall regressed towards the color category means, suggesting a reliance on color category knowledge to reconstruct previously studied color-shape pairs. To further probe the degree to which adults and children integrate their prior knowledge, they implemented and compared three computational models: the Noisy Target (Target Only) model, the Noisy Prototype (Category Only) model, and the Integrative model (see Fig. 1). The Noisy Target model assumes that information is stored in episodic memory as noisy inexact representations of true studied values (and not altered by category knowledge). The Noisy Prototype model assumes that information is stored as categorical representations of studied values. The Integrative model amalgamates the assumptions of both the Noisy Target and Noisy Prototype models and assumes that recall is combination of noisy inexact representations and categorical representations of studied values. Under this model, prior category knowledge is used to fill in the gaps when episodic traces are noisy or incomplete.

**Fig. 2:**
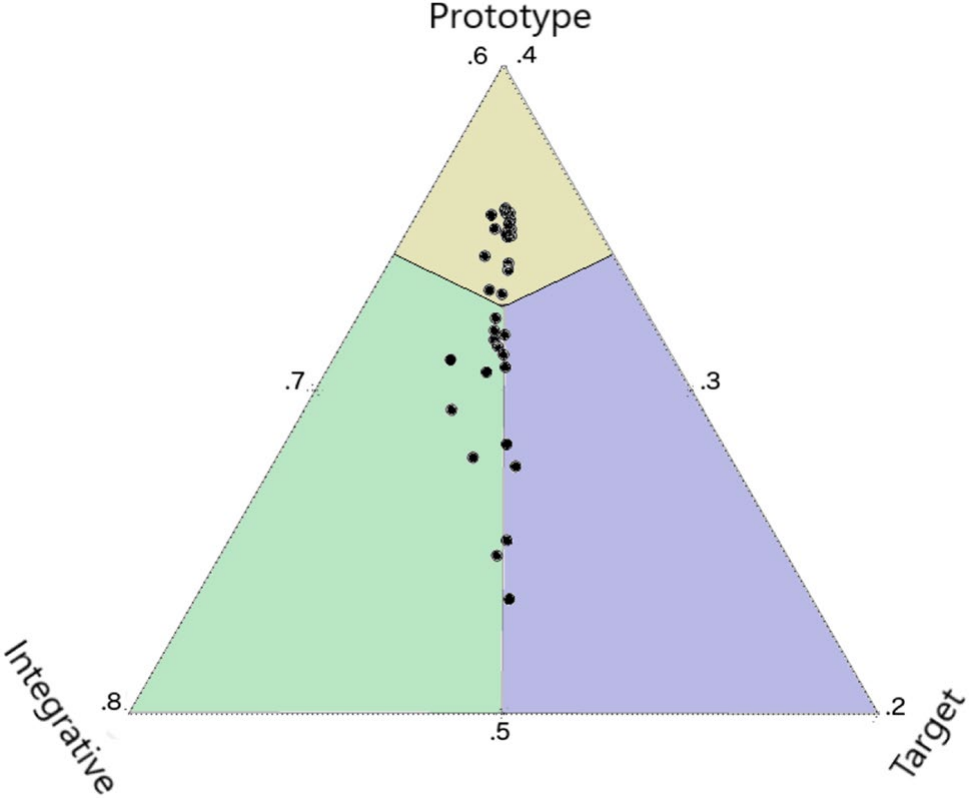
Proportion of Loglikelihood scores of individual preschooler model fits. A replicated figure from Persaud et al, 2019. Ternary plot of the proportion of log probabilities of the Integrative, Noisy Target, and Noisy Prototype models fit to each participant’s data. Data points fall within the region of the model where it is best fit. The figure has been zoomed into the approximate center of the Ternary plot for better visualization of the data

They fit the models to both adult and child recall data in the aggregate and at the individual subject level. In the aggregate, the Integrative model provided the superior fit to data from both age groups (see Table 1). At the individual subject level, unsurprisingly, majority of the adult participants were best fit by the Integrative model. However, and surprisingly, the child model fits were more diffuse with majority being better fit by the Noisy Prototype model (see Fig. 2 below). These findings suggest that the degree to which learners integrate prior knowledge and episodic traces to reconstruct a specific event varies between individuals and with age. By combining all three approaches, this work revealed the importance of looking at individual differences in age comparative studies of memory as well as formalizing and testing competing cognitive models that could explain the same behavioral patterns in memory data (regression towards the mean). In doing so, this work addresses a gap in our understanding of how prior knowledge and episodic memory tradeoff across development and when these tradeoffs are most likely to occur (i.e., as a function of the noise and uncertainty in stored representations).

**Table 1:** Frequency of model fits for preschoolers and adults

Model	Count (%)	
	Children	Adults
Integrative	11 (33.3%)	27 (79.41%)
Noisy Target	7 (21.2%)	3 (8.82%)
Noisy Prototype	15 (45.5%)	15 (45.5%)

*Note.* Table replication from Persaud et al. (2021)

## New directions: Opportunities for expansion of integrating all approaches

Persaud and colleagues (Persaud et al., 2021) provided a proof of concept of the insights that could be garnered from integrating all three approaches. The experiments and modeling approach adopted in this work could be expanded to address some open questions, as well point to future directions. In what follows, we first recapitulate at least two existing gaps in the literature on memory for expectation-related information across development and offer a new unified approach for addressing these gaps by expanding the modeling framework employed in this earlier work.

### Developmental influence of executive functioning on memory

The computational approaches employed by Persaud and colleagues (Persaud et al., 2021) were largely inspired by existing adult models of memory (e.g., Bae et al., 2015; Persaud & Hemmer, 2014) that were then applied to developmental data. While using existing adult-inspired models allows us to leverage a theoretically rich framework for investigating memory, these models often do not consider additional factors that are known to shape memory in development. For example, recent work has shown that there is a significant relationship between executive function and children’s learning (Brod et al., 2020; Bascandziev et al., 2016; Zaitchik et al., 2014), such that, in some cases, only children with strong inhibitory control abilities can learn information that is incongruent with their prior beliefs (Brod et al., 2020). It is likely that children with poor inhibitory control have more difficulty inhibiting their strong prior beliefs, leading to a greater reliance on their prior expectations, even in expectation-incongruent scenarios. Indeed, inhibitory control is a factor of executive function that has long been associated with developmental change across the lifespan (Petersen et al., 2016; Williams et al., 1999). Taken together, these findings suggest that inhibitory control may play an important role in memory for expectation-related information and potentially underscores developmental changes in performance.

It is possible that adults with limited inhibitory control will experience similar issues of inhibiting their prior beliefs when presented with expectation-incongruent information. This is an open question for future study. However, it is likely that a different executive function will play a greater role in memory for adults – i.e., attention. Past research suggests that processing expectation-incongruent information during encoding requires complex coordination between working memory and long-term memory (Foster & Keane, 2015). At a minimum, it is likely that *deliberate attention* to expectation-incongruence at encoding will be necessary for this coordinated process. Otherwise, the strategy at recall might be to fill-in noisy memories of poorly attended study events (or guess) with prior expectation-congruent beliefs (Hemmer & Persaud 2014; Persaud & Hemmer 2016). Thus, current models of expectation-related memory need to be modified with respect to the executive functions that are tapped during recall of this kind of information.

### Modeling memory for expectation-incongruent items

Second, although existing Bayesian models should theoretically capture memory for expectation-incongruent items, currently, there is little computational work characterizing memory in this context. As previously mentioned, memory for less congruent items can behave in ways not predicted by current Bayesian models. These models often treat less congruent items as members that fall on the boundaries of their associated categories (Zhang, 2022). Indeed, earlier computational work examining category learning suggests that highly incongruent items might not be stored as outlier members of their associated categories, but instead are likely to form their own separate category (Sakamoto & Love, 2004). This presents challenges for how best to flexibly represent expectation-incongruent items within existing models of memory.

In the next section, we propose a new Generative Bayesian mixture model that could capture both the impact of executive function and expectation-incongruence on episodic memory in development. We first describe the proposed model and its underlying assumptions. We then simulate recall behavior using the model, based on the hypothetical color task discussed earlier (i.e., memory for yellow versus blue banana). The simulations illustrate how varying degrees of executive functioning (e.g., attention and inhibitory control) and expectation-(in)congruence might shape recall in adults and children.

### Proposed Bayesian mixture model of expectation-related memory in development

The proposed model was developed to capture how factors of development and attention interact with expectation-congruent and incongruent information to inform recall across the lifespan. This developmentally-inspired model makes several assumptions:

1. First, this model assumes that a participant might hold multiple prior beliefs about the stimulus during an experiment with expectation-congruent and incongruent trials. One is a rigid prior belief about the real-world value of a stimulus feature (e.g., a prior belief that bananas are yellow), and another is a more flexible prior belief about the possible values of the stimulus feature in the task (e.g., a prior belief that in the task, bananas can be any color).
2. Second, the model further assumes that the prior distribution that influences recall, as assumed by the Bayesian framework, is based on a weighted mixture of the two prior beliefs (akin to hierarchical models – Hemmer & Steyvers, 2009a, 2009b; Robbins et al., 2014).
3. Third, the mixture of the two priors will depend on the participant’s executive functions and whether they are studying a congruent or incongruent target item. In children, we speculate that inhibitory control will play a role in this mixture (Brod et al., 2020), and in adults, sufficient attention to the stimulus, especially expectation-incongruent items, at encoding will influence the mixture. Because we hypothesize that inhibitory control in children will produce a similar effect on memory as attention in adults, the role of executive function can be instantiated in the model by a single parameter.
4. Finally, like most Bayesian models of memory, this model assumes that recall is a weighted combination of participants’ [mixed] prior beliefs about the studied stimulus feature and their noisy memory traces of the studied content (Huttenlocher et al., 1991; Persaud et al., 2014, 2021).

Based on these assumptions, the generative mixture model makes predictions about how inhibitory control and attention will influence memory for expectation-congruent and incongruent stimulus features. Using simulations from the proposed model, we discuss these predictions in the context of an experimental task where participants study and recall expectation-congruent and incongruent features of an object, like the object’s color.

In this task, imagine that a participant studies either a yellow banana or a blue banana and is told to recall the color. According to Assumption 1, the participant is likely to hold two prior beliefs about the object's color. One is a rigid prior belief about the true expected color of the object in the world (i.e., yellow bananas), and another is a flexible prior belief that, in this stimulus environment, objects can be relatively any color. The rigid prior belief can be represented as a distribution over the expected color category for the studied object (i.e., a distribution over yellow hue values for bananas). The flexible prior over the stimulus environment can be represented as a distribution with a high variance over the possible feature space (i.e., all colors). The high variance will allow the distribution to provide almost equal probability to all colors in the feature space.

The influence of both prior beliefs can be represented as a simple mixture of the rigid, color-specific prior and the flexible, broad prior. This mixture can be computed as: *m*(rigid prior) + 1-*m*(flexible prior), where *m* represents the weight given to each prior in the mixture. According to Assumption 3, the weight *m* will be governed by the participants' level of executive functioning – attention (i.e., adults) and inhibitory control (i.e., children) as well as whether the target is expectation-congruent or incongruent.

After computing the mixed prior, the rest of the model follows a simple rational model of memory (Huttenlocher et al, 1991; Persaud & Hemmer, 2014). The stimulus color values are assumed to be drawn from the mixed prior distribution reflecting the stimulus environment. Studying the color is assumed to produce noisy memory traces of the color. Given the participant’s mixed prior expectations about study values and their noisy memory representations, the goal for the participant at test is to recall the study value using their noisy samples and their prior expectations. Bayes' rule gives a principled account of how participants might combine their noisy memory traces with the mixed prior to reconstruct the studied stimulus.

**Fig. 3:**
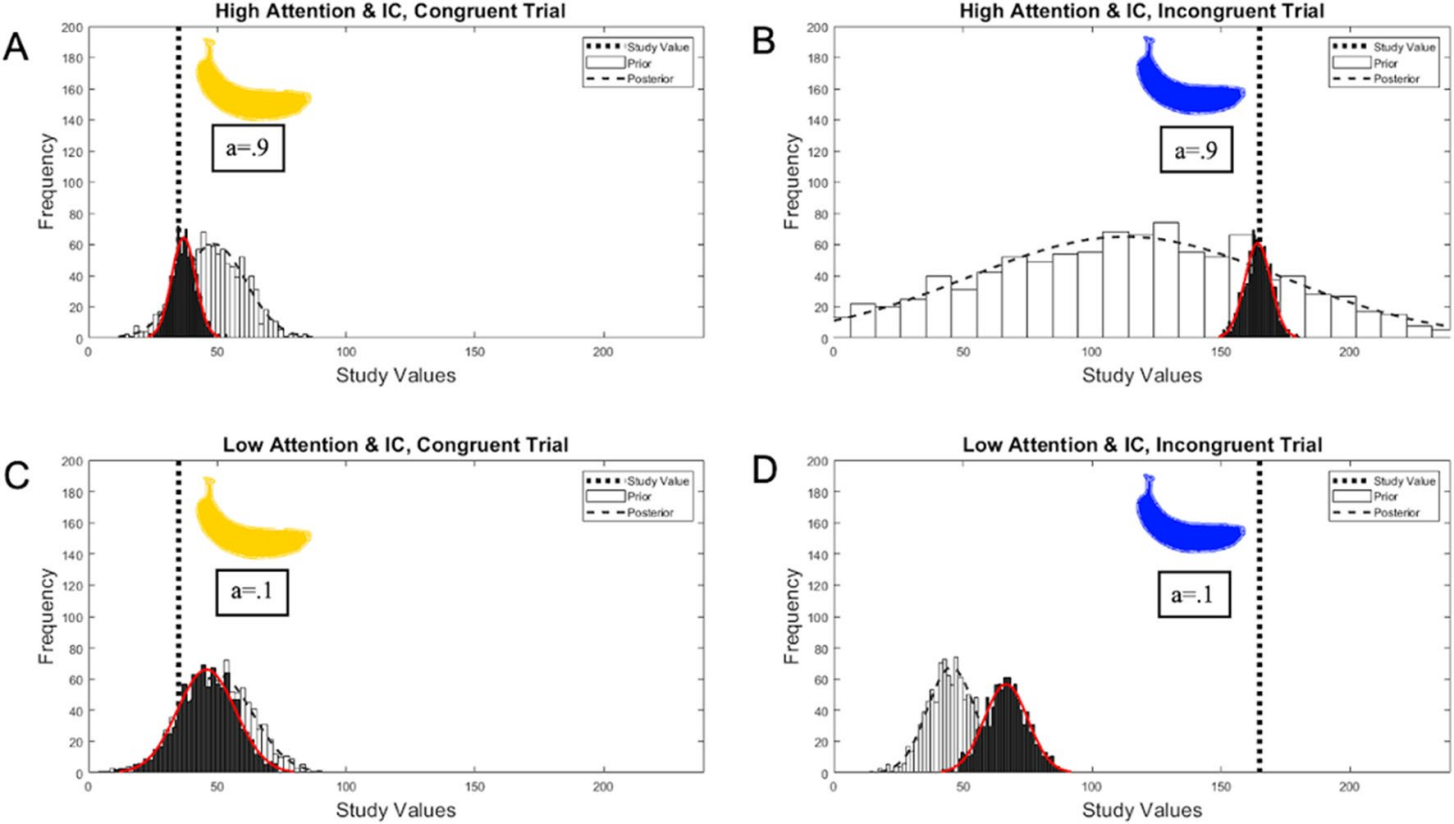
Simulated model predictions of the systematic biases that would result in recall as a function of expectation-congruency and executive functions. **Panel A:** the prior and posterior distributions for expectation-congruent trials when attention and inhibitory control are high. **Panel B:** the prior and posterior distributions for expectation-incongruent trials when attention and inhibitory control are high. **Panel C:** the prior and posterior distributions for expectation-congruent trials when attention and inhibitory control are low. **Panel D:** the prior and posterior distributions for expectation-incongruent trials when attention and inhibitory control are low

Several predictions fall out of the simulations of the proposed mixture model. If a participant has good attention and inhibitory control and has studied an expectation-congruent color, the mixed prior will give more weight to the rigid, color-specific prior (Fig. 3, panel 1). As such, recall might closely resemble the expectation-congruent study color. If a participant has good attention and inhibitory control and has studied an expectation-incongruent color, the mixed prior will give more weight to the flexible, broad prior (Fig. 3, panel 2). Given the noise on the flexible prior, recall will more closely resemble the noisy memory traces, producing a recall value close to the studied expectation-incongruent color. If a participant has insufficient attention and inhibitory control, regardless of whether they studied an expectation-congruent (Fig. 3, panel 3) or incongruent color (Fig. 3, panel 4), the mixed prior will give more weight to the rigid, color-specific prior. In both cases, recall will more closely resemble the rigid prior. This is meant to capture the intuition that if an adult is not paying attention during encoding, they are more likely to guess with their prior expectations. Similarly, if a child has insufficient inhibitory control, they will have difficulty ignoring their color-specific prior belief, and they, too, might guess based on their strong prior expectations.

### What do we stand to learn from the proposed model

The proposed model brings together behavioral, computational, and developmental approaches to further our understanding of how prior knowledge and expectations influence episodic memory across the lifespan. For developmental contexts, this new model allows us to evaluate whether and how changes in inhibitory control and prior knowledge drive differences in memory processes across development. For adult contexts, this new model provides an avenue to address disparate findings in the literature on how expectation (in)congruence impacts memory by evaluating the contribution of attention at encoding. This attention might depend on several factors such as the degree to which the information coheres with or violates an expectation and how well entrenched the expectation is at the onset of study. More broadly, the model can capture the strength of expectation, relate that strength to attention, and evaluate the impact of both on memory.

For computational approaches, this new model offers a way to augment existing models to better fit and evaluate developmental data. It also tests a theory of how expectation-incongruent items might be represented (as a more flexible prior category) within a single model of memory. The addition of this new component might allow existing approaches to better characterize memory for incongruent items (see Zhang, 2022, for a discussion). Finally, the proposed model is a process model. As such, it not only predicts memory outcomes, but also posits how expectation-related information might be represented in the mind, the process of encoding and retrieving expectation-related information from memory, how this process is influenced by executive functions and congruence, and most impactfully, how this process changes across development.

### How to test the predictions of the model

There are several interesting avenues to test the proposed model. Here we discuss just a few. First, the model pinpoints two specific executive functions that might shape memory across children and adults. Yet, it is possible that other executive functions may affect memory performance. For instance, Foster and Keane (2019) theorize that processing expectation-incongruent information relies on working and long-term memory to help reconcile the incongruency and facilitate storage. As such, it is possible that working memory capacity could also contribute to developmental differences in memory, especially for congruent and incongruent items. Furthermore, while the proposed model predicts the influence of executive function on memory, it is crucial to acknowledge that episodic memory abilities, in general, are dynamic across development (Lloyd et al., 2009) and could affect memory for expectation-related information. By gathering data from different age groups that vary in their episodic memory maturation (along with executive function assessments), we can leverage the proposed model to understand tradeoffs between prior knowledge and episodic memory across developmental age groups.

In addition, it is worth noting that the attention that influences memory in the model can be conceptualized in multiple ways. This attention parameter could reflect general attentional capacity which might play a role in recall. It could also reflect a stimulus driven attention that will undoubtedly impact memory, especially in contexts of expectation-incongruence (Foster & Keane, 2019). As such, it is possible that one measure of attention (e.g., a task that forces specific attention to expectation-incongruence) will better align with the predictions of the model compared to another more generalized measurement of attention. Importantly we can utilize this proposed model to explore how different operationalizations of a single construct (e.g., attention) impact the predicted memory outcomes. This will help us gain clarity on the exact executive function facilities that matter for the reconstructive memory process across the lifespan, particularly for instances of expectation-incongruence.

Furthermore, the strength of an individual’s prior expectation is likely to vary across stimulus domains with some domains being much more entrenched and well-established (e.g., beliefs about biology and intuitive physics) compared to others (e.g., category schemas). The strength and type of prior knowledge might determine the weighting of the prior mixture in the model. As such, we can use the proposed model to test the impact of varying priors and stimulus domains by applying the model to data across diverse domains of knowledge. This endeavor will illuminate how much differences in stimulus domains contribute to mixed findings in the literature.

Finally, we can explore how well the proposed model captures the impact of expectations on memory by manipulating the level of a studied item’s expectation-congruence. For instance, there is some evidence to suggest that adults better remember information that is very surprising relative to information that is somewhat surprising and expected (Foster & Keane, 2019). Differences in the level of expectation-incongruence might inform both the attention and flexible prior parameters in the model. Therefore, we can use the current model to explore how varying levels of incongruence impacts memory for expectation-related information**.**

Taken together, future behavioral studies with children and adults are warranted to test the assumptions and predictions of the generative model. These studies would need to include measures of attention and inhibitory control capacities as well as episodic memory for expectation-congruent and incongruent features. Also, while the proposed model simulations utilized continuous distributions to reflect the fact that color is a continuous feature value, the model can be augmented to capture memory for different types of stimulus domains by changing the distributional forms of the model parameters.

## General discussion

Within the past few decades, research has substantially improved our understanding of the influence of prior knowledge and expectations on memory. This work has employed three often isolated (but not mutually exclusive) methodological focuses: strictly behaviorally motivated investigations with adult populations, computational cognitive modeling, and cognitive development. Importantly, each approach has produced fruitful results, *somewhat* independent of the others. For example, behavioral approaches have facilitated the development of critical data to test and revise theories of how prior knowledge and expectations influence memory performance. Computational approaches have extended beyond standard means of analyses to provide principled accounts of the processes that underlie the memory system and evaluate how different factors contribute and interact during memory processes. Developmental approaches have capitalized on the rich source of variability in children’s knowledge and memory ability to refine theories of memory and further understand the interaction of memory and prior knowledge.

While much has been learned using each of these approaches, there is still much left to be discovered as mixed findings on memory for expectation-related information are in abundance and open questions persist. Here we point out that integrating adult behavioral, computational, and developmental approaches might be a good method for reconciling some of the mixed findings and addressing open questions and in general moving the study of episodic memory and expectations forward. As proof of concept, we have discussed a previous study that integrates all three approaches to address significant gaps in the literature (Persaud et al., 2021). This investigation has helped shape the current discussion of the role of prior knowledge in memory. Combining all three approaches has provided critical insight into the underlying processes of the memory system and points to a particular mechanism that might account for some developmental shifts in memory performance. Importantly, this integrative framework allows us to explore further questions regarding the functionality and constraints of the adaptive memory system, more broadly. We emphasize that while we have learned quite a bit from this one study, this work only begins to scratch the surface of the gains that can be acquired from integrating across all three approaches, and especially for combining computational modeling and development. Building on this work, we propose a new computational model that posits how additional behavioral and developmental factors might work in concert to inform interactions between prior knowledge and episodic memory. Simulations from this model reveal explicit predictions about these complex interactions that can be tested in future behavioral investigations of memory across development.

Finally, while we lobby for integrating all three approaches to further our understanding of the relationship between expectations and memory, we do concede that these are not the only approaches that are fruitful and necessary for this line of investigation. As previously mentioned, there are critical studies from the domain of cognitive neuroscience that provide important theoretical frameworks and sophisticated research tools (e.g., FMRI, TMS) to examine the relationship between prior knowledge, expectations, and memory. Corroborating evidence across multiple methodologies presents the strongest case for evaluating cognitive questions of interest. Nevertheless, here we lobby for computational and developmental approaches for exploring episodic memory for several reasons. Computational approaches are becoming increasingly popular and accessible as a means of sharpening the analytic toolkit of cognitive researchers (see McClelland, 2009, for a similar argument). Computational modeling is a tool that is attainable both in terms of training (e.g., via workshops or textbooks) and financially, as many models can be carried out on standard computers. While of course there are other methodologies to consider and even more to be discovered, integrating adult behavioral, computational, and developmental approaches to understand the processes and mechanisms that underlie episodic memory is a good place to start.

## Conclusion

Bridging together adult behavioral, computational, developmental approaches can further inform our understanding of the goals and processes that underlie episodic memory. Together, they can shed light on the contexts and domain dependencies that govern when, how, and why prior knowledge and expectations influence memory. Finally, integrating over all three approaches provides a sophisticated toolkit for testing and revising our theories of memory processes and is a transformative way to move the field of prior knowledge, memory and learning forward.

## Footnotes

1 Here we use *expectations* as a broad term to refer to any kind of knowledge and expectation an individual has about study memoranda. These expectations can be pre-existing or be learned over the course of a task. They can also take the form of schema knowledge, stereotypes, scripts, category knowledge and expectations, category rules, etc. While these instances may refer to different kinds of information, we use expectations as a catch-all term. In addition, although expectations are described differently and reflect different kinds of information, there appears to be synergy in understanding how they impact memory across various fields (e.g., social stereotypes – Stangor & McMillan, 1992; Sherman & Frost, 2000; event memory – Greve et al., 2019; van Kesteren et al., 2010; category learning – Sakamoto & Love, 2004; episodic memory – Persaud et al., 2021; visual working and long-term memory – Bae et al., 2015). Thus, in this paper, we use the word expectations, in a general sense, to reflect different kinds of information that influence memory.

2 Similar arguments for integrating approaches, particularly leveraging developmental data to test and expand computational frameworks to understand cognition more broadly have been recently advanced (responses to Leider & Griffiths, 2020, resource-rational analysis; Bejjanki & Aslin, 2020; Persaud et al., 2020). Here we push to put this new theoretical treatment into practice, specifically as it relates to episodic memory given the prime opportunity integrated approaches create for reconciling mixed findings and addressing open questions regarding the nuanced relationship between prior expectations and memory.

3 We use the term *general* to refer to models that do not explicitly parameterize prior knowledge and expectations.
